# Excerpts from Letters Concerning Package Library

**Published:** 1915-01

**Authors:** 


### Excerpts From Letters Concerning Package Library.

DEAR MRS. DANIEL: Enclosed find $1.00 for the Belgian physicians' fund. If only 50 per cent of the physicians of Texas will each contribute this small amount, the aggregate sum will prove a great blessing to our sorely afflicted colleagues in stricken Belgium. I heartily endorse your package library system, advocated in December issue of the TEXAS MEDICAL JOURNAL. You will, doubtless, be urged to push the scheme to completion.

With best wishes of an old subscriber to the redoubtable "Red Back," I am,

Sincerely yours,

A. GARWOOD, M. D.

---

MY DEAR MRS. DANIEL: I think your plan for a Package Library System is one of the most sensible ideas conceived by anyone, and can be especially beneficial to the busy practitioner. I will be only too glad to take advantage of it.

J. C. HOLMAN, M. D.

---

*Mrs. F. E. Daniel, Austin, Texas.*

DEAR MRS. DANIEL: I believe that you will be able to build up a very valuable library, which will be of great service to the profession. In fact, my belief is that it would become so valuable in time that the physicians of the State would pay a rental for the privilege of reading such classified matter when they are looking up some particular subject.

J. S. LANKFORD.

MRS. F. E. DANIEL: I think your Package Library plan a grand idea, and that it will result in much good.

J. R. EVANS.

---

MY DEAR MRS. DANIEL: I am very much in favor of the Package Library System, and pledge my support in every possible way.

Yours very truly,

J. B. MCKNIGHT,

Superintendent State Tuberculosis Sanitarium.

---

DEAR MRS. DANIEL: I am so glad to know that you are not only interested in this idea of a circulating medical library, but are actually about to put it into effect. The one thing that I think we doctors in this far off section need most is a package library system. I not only most heartily endorse the plan, but congratulate you on your spirit and energy in attempting to give us so many benefits through your labor.

Sincerely yours,

C. S. VENABLE.

My Dear Mrs. Daniel: Thank you for reminding me of arrears. I would not miss the "Red Back" for anything. So long as I am able to subscribe for any journal, I shall place the "Red Back" first on the list.

Best wishes for your success.

Mrs. Sadie Haley.

---

Dear Mrs. Daniel: I think you are conducting the most interesting department of medicine to be found in any medical journal in the country. I refer to your Department of Eugenics. I certainly wish you continued success in the work and feel that you are more than "making good."

Respectfully,

W. L. Crothwait, M. D.

---

May I congratulate you on "The Doctor's Prayer" in the December "Red Back." It is a poem of surpassing beauty and tenderness, and will no doubt be greatly appreciated. Such meritorious editorials will soon achieve widespread fame for your Journal. I heartily approve your Package Library System.

M. D.

---

Dear Madam: I approve your idea greatly, and think it would be a great help. Furthermore, whatever the "Red Back" undertakes is always a good thing and successful.

Wishing you a happy, prosperous New Year. With best wishes, I am,

Yours truly,

Leo. J. Peters, M. D.

